# Electrospun Anion-Conducting Ionomer Fibers—Effect of Humidity on Final Properties

**DOI:** 10.3390/polym12051020

**Published:** 2020-05-01

**Authors:** Manar Halabi, Meirav Mann-Lahav, Vadim Beilin, Gennady E. Shter, Oren Elishav, Gideon S. Grader, Dario R. Dekel

**Affiliations:** 1 The Wolfson Department of Chemical Engineering, Technion−Israel Institute of Technology, Haifa 3200003, Israel; manarh@campus.technion.ac.il (M.H.); meiravml@technion.ac.il (M.M.-L.); vadimbe@technion.ac.il (V.B.); shter@technion.ac.il (G.E.S.); orene@campus.technion.ac.il (O.E.); 2 The Nancy & Stephan Grand Technion Energy Program (GTEP), Technion, Israel Institute of Technology, Haifa 3200003, Israel

**Keywords:** electrospinning, fibrous morphology, relative humidity, polymer fibers, ionomer

## Abstract

Anion-conducting ionomer-based nanofibers mats are prepared by electrospinning (ES) technique. Depending on the relative humidity (RH) during the ES process (RH_ES_), ionomer nanofibers with different morphologies are obtained. The effect of relative humidity on the ionomer nanofibers morphology, ionic conductivity, and water uptake (WU) is studied. A branching effect in the ES fibers found to occur mostly at RH_ES_ < 30% is discussed. The anion conductivity and WU of the ionomer electrospun mats prepared at the lowest RH_ES_ are found to be higher than in those prepared at higher RH_ES_. This effect can be ascribed to the large diameter of the ionomer fibers, which have a higher WU. Understanding the effect of RH during the ES process on ionomer-based fibers’ properties is critical for the preparation of electrospun fiber mats for specific applications, such as electrochemical devices.

## 1. Introduction

Electrospinning (ES) is broadly applied to generate nanofibers from a wide range of materials, including polymers, metals, ceramics, and composites [1,2,3,4,5]. This technique [6,7,8,9,10] allows control of the fibers’ morphology and diameter, which play important roles in their final applications. Solution properties such as precursor concentration, polymer molecular weight, viscosity, solvent characteristics as well as process conditions greatly affect the electrospun fibers [7,8,11,12,13,14]. Such nanofibers have potential applications in the biomedical [15,16], energy [17,18], and other fields. The electrospinning process often involves usage of organic solvents, which are toxic, expensive, and considered environmentally unfriendly. This motivated the researchers to develop the “green process”, which is a more eco-friendly ES process that uses aqueous polymer solutions [19,20]. In addition, the high viscosities of the polymer solutions require using low polymer concentrations, thereby limiting the utilization of the ES process due to the large amounts of organic solvents needed. The “green process” overcomes these limitations, allowing higher polymer concentrations in water for more effective and productive ES procedure [21].

The influence of environmental conditions during electrospinning on the morphology of polymer fibers has been investigated [11,22,23]. For example, the relative humidity during ES, RH_ES_, plays a critical role for the formation of “porous morphology” [24]. Casper et al. [12] studied the effect of increasing RH_ES_ and varying polystyrene (PS) molecular weight (MW) on the pore size distribution of the electrospun fibers. They found that raising the RH_ES_ increases the number of pores and their diameter. Higher MW leads to larger pores on the fibers’ surface. The effect of changing RH_ES_ and solvents ratios on the surface morphology of electrospun PS fibers was reported elsewhere [25,26]. A study demonstrated the effects of solution’s viscosity and RH_ES_ on fibers’ morphology and claimed that monitoring these two parameters facilitated fibers’ architecture control, which changed from beaded fibers to smooth uniform fibers [23]. In addition to examining the external morphology, Pai et al. [27] presented porous structures within electrospun PS fibers while electrospinning at RH_ES_ in the 24–43% range. Based on TEM analysis, the void fraction of electrospun PS in DMF was about 30%. It was shown that the internal pores have a significant effect on the mechanical, optical, as well as electrical properties of the fibers. 

Although electrospinning of (inert) polymers has been extensively reported, this process was scarcely studied for the case of ionic polymers, usually called ionomers [28,29,30,31,32,33,34,35,36,37]. While some proton-conducting ionomers have been the base of first attempts to produce electrospun proton-conducting fiber mats [38,39,40,41,42,43], only a few studies can be found on electrospun anion-conducting fiber mats [17,44,45]. 

In this work, we investigate the electrospinning process of an anion-conducting ionomeric material. We specifically focus on an interesting branching phenomenon that is observed in anion-conducting fibers that were electrospun under 30% RH_ES_. According to a study by Yarin et al. [46], branching occurs when the static undulations of a cylindrical jet become unstable at the sites of the highest local curvature, where secondary jet branches are ejected from the primary jet. This phenomenon was also explained by Tan [47]. The branching effect had been demonstrated on polymer fibers [48] and piezoelectric fibers [49]; however, all the studies mentioned above were done using nonconducting polymers. The branching effect can be leveraged to drastically increase the surface area of fiber products; nevertheless, it is unclear what governs this effect in conducting polymers and whether they are as sensitive to the environmental conditions during the electrospinning process as nonconductive polymers. Moreover, due to the ionic character of the anion-conducting ionomers, it is especially interesting to investigate the effect of the ES process on their final properties.

The goal of this article is to investigate the effect of RH_ES_ on the electrospun anion-conducting polymer nanofibers’ morphology as well as fibers’ mat properties. The effect of RH_ES_ on the branching of anion-conducting polymer is presented and a qualitative model explaining this effect is suggested. Understanding the relationship between RH_ES_ and the structure, WU, and anion conductivity of the electrospun ionomer nanofibers would enable us to achieve fiber mats with tailored and improved properties for their numerous applications in electrochemical devices. Such fiber mats can be used, for instance, in advanced anion exchange membrane fuel cells (AEMFCs) [50,51,52,53,54] as a catalyst bed as well as a membrane.

## 2. Experimental

Anion-conducting ionomeric material (FAA-3 in its Br^–^ form) was purchased from Fumatech BWT GmbH, Germany. FAA-3 is a quaternary ammonium functionalized aromatic PPO (Poly (p-phenylene oxide))-based polymer. The solvent used for preparing the ES precursor solutions was N,N-dimethylformamide (DMF) (Bio-Lab ltd., Jerusalem, Israel). Precursor solutions were prepared by dissolving the anion-conducting ionomer in DMF with ionomer concentration of 30 wt %. The solutions were magnetically stirred for 2 h at room temperature until complete dissolution of the ionomer. 

The ionomer solutions were electrospun in an electrospinning machine—NS24, (Inovenso, Istanbul, Turkey), in an environmentally controlled chamber. Precursors were loaded into a 5-mL syringe and fed to the ES system at a flow rate of 0.3 mL h^−1^ for 6 h. The tip to collector distance was 14.5 cm. A positive charge of 20 kV was applied on the needle, and a negative charge of 3 kV was applied to a flat collector. The flat collector size is 130 mm by 370 mm, it moved back and forth 60 mm along the long axis at a speed of 10 mm s^−1^ to ensure fiber deposition homogeneity. The chamber’s temperature was set constant at 24–25 °C, and the RH_ES_ was varied in the range of 20%–50%. 

The obtained ionomeric mats were dried in a vacuum oven at 40 °C for 16 h to remove the residual solvent. The morphology of the nanofibers was characterized by high-resolution scanning electron microscopy (HR-SEM, Ultra Plus, Zeiss, Switzerland) at a magnification range of ×1000–10,000. The diameter of the electrospun fibers was obtained by HR-SEM images analysis of three different areas on ionomer mats deposited on carbon conductive tape. The fibers’ diameter distribution was calculated by a statistical analysis of at least 30 data points from each sample.

The WU of the electrospun ionomer samples was measured using a VTI SA + instrument (TA Instruments, New Castle, DE, USA), using protocols detailed elsewhere [55,56,57,58]. In brief, each electrospun ionomer sample was dried in situ for a maximum of 60 min at 50 °C and RH close to 0%. After that, the temperature was set constant at 40 °C and the RH was then raised from 10% to 90% in intervals of 20%. Each RH step was maintained until the sample weight reached equilibrium (WU changes smaller than 0.001 wt % in 5 min). The temperature during the WU measurements was kept at 40 °C. Values of WU were calculated using Equation (1) [55,59], where the “wet” and “dry” weight (*W*_(*wet*)_ and *W*_(*dry*)_, respectively) were measured at the end of each equilibrium step and at the end of the initial drying step, respectively:

(1) $$WU=\frac{W\left(wet\right)-W\left(dry\right)}{W\left(dry\right)}\times 100\%.$$

The WU kinetics were also determined, by measuring the mass change of the ionomer mat electrospun sample as a function of time at every RH step. The characteristic time constant, *τ*, was calculated by fitting the experimental data with the following equation [55,60]:

(2) $$\frac{W_{t}-W_{0}}{W_{\infty }-W_{0}}=\frac{M_{t}}{M_{\infty }}≅1-exp\left(-\frac{t}{\tau }\right),$$

where *W_t_* is the mass of sample at time *t*, *W*_0_ is the mass at the beginning of the RH step, *W_∞_* is the mass of membrane at equilibrium state, *M_t_* is the mass gain at time *t*, and *M_∞_* is the mass gain of the ionomer mat at equilibrium.

Bromide anion conductivity measurements were conducted on the ionomer mats electrospun at different RHs. These measurements were made in a conductivity chamber of an MTS-740 ionic conductometer (Scribner Inc., Southern Pines, NC, USA) using the protocol detailed elsewhere [58]. The bromide anion conductivity was calculated using the ionomer mat resistance measured with a 4-point probe cell in a sealed, thermally insulated chamber under continuous N_2_ gas conditioned to the desired humidity. The ionomer mat samples were first equilibrated at 40 °C at 90% RH for 1 h, the RH was then decreased from 90% to 10% in intervals of 20%, then back from 10% to 90%, following the procedure reported elsewhere [61]. Resistance values were measured perpendicular to the fiber mat, in the through-plane (TP) direction. The TP resistance, R, was measured by impedance spectroscopy using PSM1735 Frequency Response Analyzer (Newtons4th Ltd., Leicester, UK). The TP conductivity was then calculated as [61,62]

(3) $$\sigma_{TP}=\frac{d}{A\cdot R},$$

where *d* is the mat thickness, *A* is the cross-sectional area through which the current passes (0.5 cm^2^), and *R* is the measured resistance.

The velocity of the jet was calculated using the model reported by Ding et al. [63]. This model is based on the mass conversation of the polymer in the fiber. While the jet is elongated under different ES parameters and times, the volume of polymer along the fiber length must equal the volume of polymer that was consumed. Therefore, the model considers the diameter of the jet. The velocity is calculated by

(4) $$v_{1}=\frac{Q_{s}}{S_{1}\cdot t},$$

where *Q_s_* stands for the consumption of spinning solution (mL), *S*_1_ is the cross-sectional area of the fiber (µm^2^), and *t* is the electrospinning time (h).

## 3. Results and Discussion

### 3.1. Ionomer Fibers Morphology

**Figure 1:**
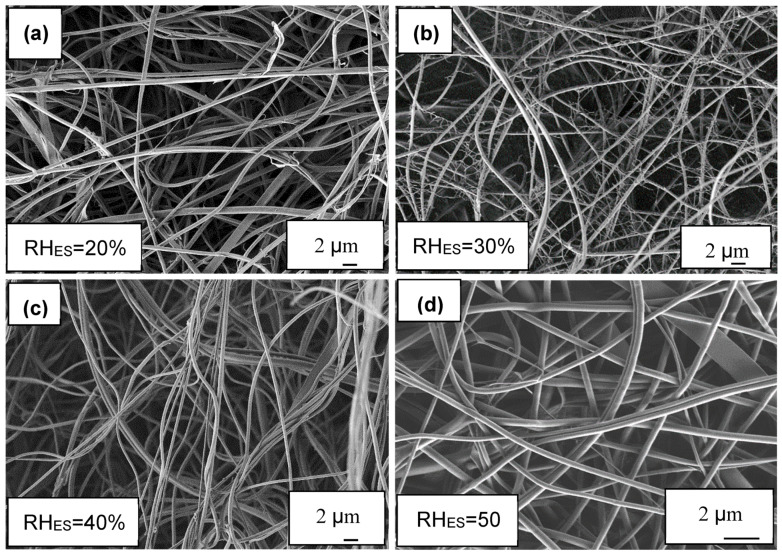
SEM images of ionomer fibers electrospun at RH_ES_ range of 20%–50%. All SEM images taken at ×5000 magnification. (**a**) 20% RH_ES_, (**b**) 30% RH_ES_, (**c**) 40% RH_ES_ and (**d**) 50% RH_ES._

**Figure 2:**
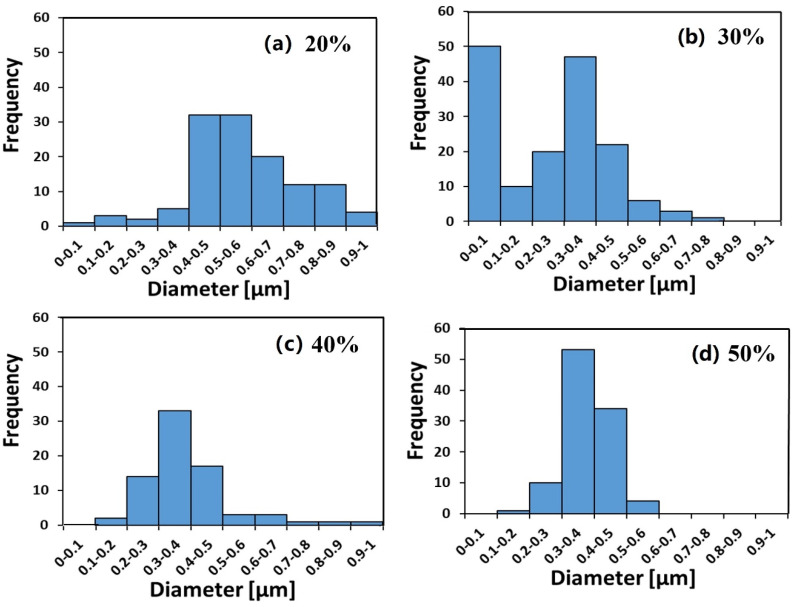
Fibers’ diameter distribution of ionomer fibers electrospun under a relative humidity (RH_ES_) range of 20%–50%. (**a**) 20% RH_ES_, (**b**) 30% RH_ES_, (**c**) 40% RH_ES_ and (**d**) 50% RH_ES._

The ionomer fibers electrospun at RH_ES_ = 20% were mostly flat belts. This can be rationalized by the formation of an early skin on the fibers due to fast evaporation at RH_ES_ = 20%. The skin prevents uniform fiber shrinkage and eventually collapses into a belt (Figure 1a). A similar effect has been observed and modeled in our work on other fiber materials [64,65]. At RH_ES_ = 30%, a more significant branching effect was observed (Figure 1b); while at RH_ES_ = 40% and 50%, the fibers were smooth and cylindrical (Figure 1c,d) without any visible branching. This observation of the electrospun ionomer branching effect is further confirmed by measuring the fiber diameter distribution (Figure 2).

The diameter distributions at RH_ES_ = 20%, 40%, and 50% are monomodal (Figure 2a,c,d), whereas at RH_ES_ = 30%, a bimodal diameter distribution can clearly be seen in Figure 2b. The diameter of the branches (small fibers) is in the 80–180 nm range.

**Figure 3:**
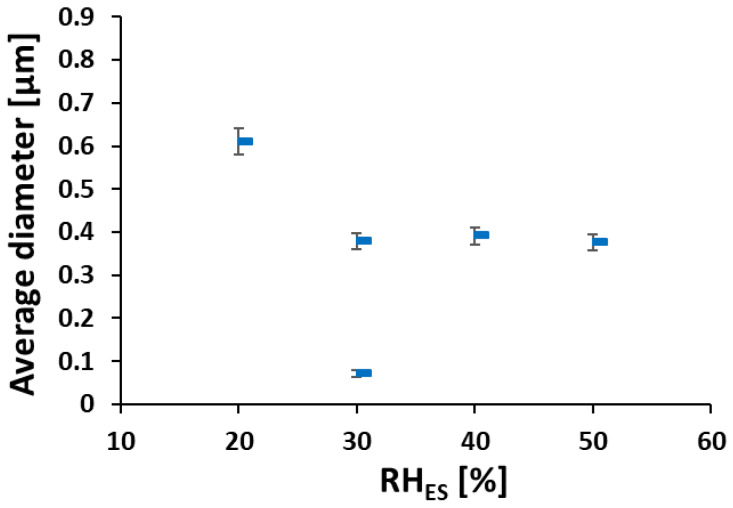
Fibers’ average diameter vs. RH_ES_ (calculated from data in Figure 2).

As RH_ES_ increased from 20% to 30%–50%, the main average fiber diameter decreased from ~600 nm to a constant value of ~400 nm (see Figure 3). The larger width of the fibers electrospun at RH_ES_ = 20% may be due to their flat morphology, where the original cylindrical fiber collapsed into a belt, thus spreading over a larger width. The bimodal diameter distribution at 30% RH_ES_ can be explained by the branching mechanism, due to excess charge in unstable areas on the primary cylindrical jet [48,49]. Although some small ionomer fibers are also observed during ES at RH_ES_ = 20%, the diameter distribution of the electrospun ionomer fibers is not bimodal. 

**Figure 4:**
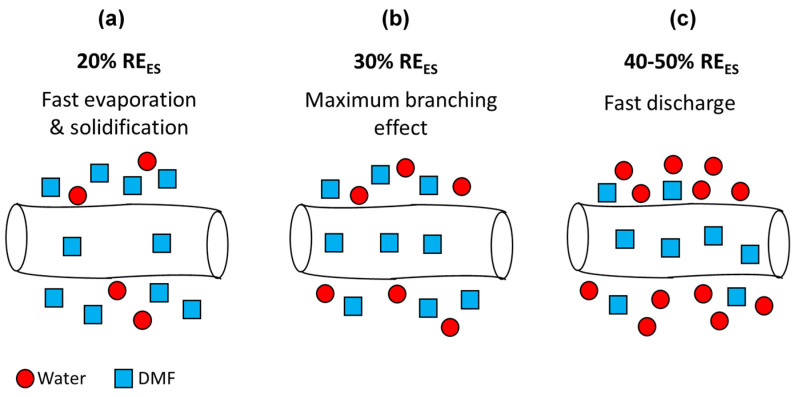
Scheme of electrospun fibers during their flight towards the collector at different RH_ES_: (**a**) 20%, (**b**) 30%, and (**c**) 40–50%.

Depending on the hydrophobic or hydrophilic nature of the polymer [66], the RH can either increase or decrease the nanofibers’ diameter. When hydrophilic polymers such as polyvinylpyrrolidone and polyethylene oxide are electrospun at higher RH, the polymers solutions solidify more slowly due to slower evaporation rate [67]. This gives rise to a smaller diameter due to further stretching of the fiber. Another effect that plays a role here is that at a higher RH, the electric charges on the fibers’ surface can discharge to the surrounding water vapor more easily. Thus, the charge on the fiber is reduced, hence the attraction to the collector and stretching force are reduced, which in turn leads to larger fiber diameters [26]. In the present case, ES at low RH_ES_ (20%) caused fast evaporation of DMF, giving rise mostly to flat fibers with an average width of 600 nm. Above RH_ES_ = 20%, fast evaporation of the solvent is prevented, more stretching occurs, and the cylindrical cross-section is maintained. At 40% and 50% RH_ES_, the discharging effect becomes more dominant, thus we do not see an additional decrease in fibers’ diameter below 400 nm as shown in Figure 4.

The two trends described above, involving solvent evaporation and discharging, occur simultaneously and have opposing effects. The maximal branching phenomena at 30% RH_ES_ has not been observed before. It is not clear why branching is subdued at lower RH and eliminated at higher RH. Baumgarten et al. [68] investigated the effect of surrounding gas during ES on fibers’ morphology. Electrospinning in Freon atmosphere gave rise to a branching effect. However, ES in air at identical conditions (15 kV and 75 TCD) did not show any branching. This difference was attributed to the higher breakdown voltage of Freon compared to air. We believe that the lower breakdown voltage of the humid atmosphere during ES at high RH_ES_ (40–50%) causes a discharge of the electric charges on the fibers’ surface to the surrounding water vapor. This effect reduces the surface charge concentration at the unstable points; thus, branching does not occur.

### 3.2. Water Uptake

**Figure 5:**
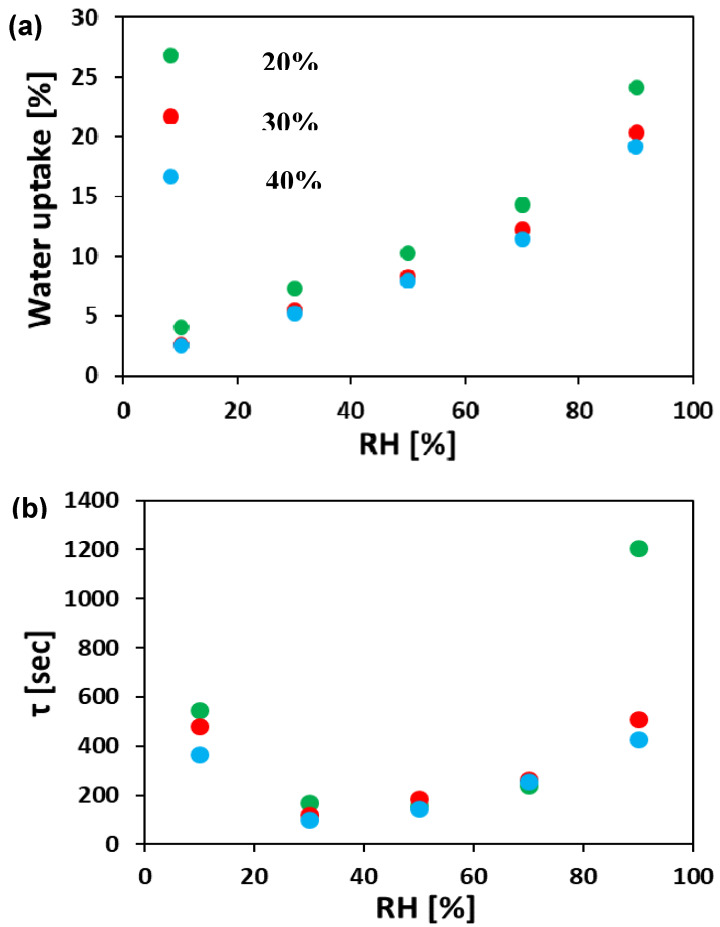
(**a**) Water vapor absorption isotherms and (**b**) characteristic time constant, τ, for electrospun ionomer mats. All tests were done at 40 °C. RH—relative humidity.

It can be seen (Figure 5a) that the WU increases with RH for all the ionomer mat samples. Over the whole range of RH values, the ionomer mat sample prepared at RH_ES_ = 20% has higher WU compared with those electrospun at higher RH_ES_. The ionomer mat samples prepared at RH_ES_ = 30% and 40% have almost the same WU, even though the sample prepared at RH_ES_ = 30% has a bimodal fiber distribution. The reason for this is that the weight fraction of the small diameter segment in the bimodal sample is 1/16 of the larger diameter segment. (The average diameter of the fibers in the bimodal sample are 50 and 400 nm, while the ratio of small vs. large ionomer fibers per unit area is 4). Hence, water absorption on the small fibers does not contribute significantly to the overall WU.

The characteristic time constant, τ, was calculated from Equation (2), as described in the experimental section. Figure 5b summarizes the resulting τ for all samples. The electrospun ionomer mats had a relatively large τ at 10% RH (350–550 s). As RH is increased, τ decreases to a minimum at RH (30–50%) and then increases (τ = 400–1200 s) at higher RH. The minimal value of τ in the 30%–50% range of RH indicates that the fastest WU kinetics in these materials occurs in the mid-RH region. This is consistent with the trends found for WU studies of different nonporous anion-conducting membranes [55,64]. 

The high value of τ at low RH can be rationalized by the presence of smaller water content in the gas phase, thus resulting in a lower flux of water molecules towards the surface. The faster WU kinetics (lower values of τ) in the mid-range RH (30–50% RH) can be explained by the larger initial water content within the ionomer fibers, which lowers their density and the larger flux of water from the gas phase at high RH. This combination makes it easier for additional water molecules on the surface to diffuse into the ionomer fiber. When the RH is the highest (90%), the fibers are nearly saturated and hence it is more difficult for additional water molecules to diffuse through the fiber, resulting therefore in higher τ values.

### 3.3. Anion Conductivity 

**Figure 6:**
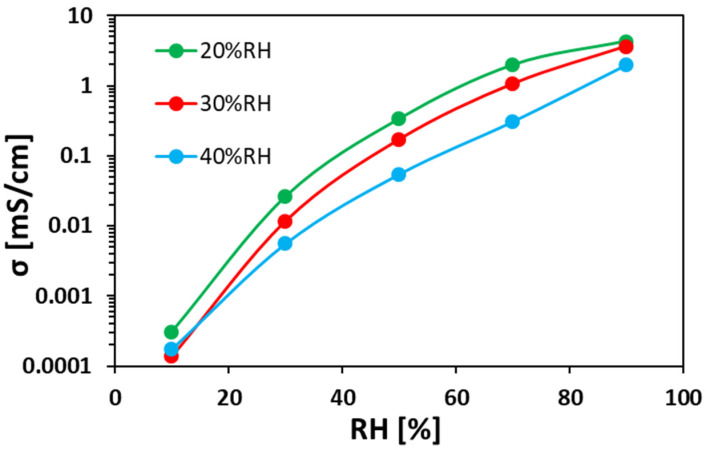
Bromide TP anion conductivity at 40 °C vs. RH for electrospun ionomer fibers prepared at different RH_ES_.

Figure 6 shows the bromide TP anion conductivity at 40 °C as a function of RH for ionomer fibers prepared at different RH_ES_. As can be seen, Br^–^ anion conductivity significantly increases with increasing RH. As can be seen, the anion conductivity of the electrospun ionomer mats made at lower RH_ES_ are higher than mats electrospun at larger RH_ES_. For instance, at 70% RH, the Br^–^ TP anion conductivity of electrospun mat under 20% RH_ES_ is 6 times higher than that of a mat prepared at 40% RH_ES_.

A clear relation can be concluded from the results in Figure 5a and Figure 6. In both figures, the order is kept: the electrospun ionomer mat at 20% RH_ES_ has the highest WU and highest anion conductivity at RHs above 50%, compared to both mats prepared at 30% and 40% RH_ES_. This indicates that higher WU and density promote faster ion transfer within the ionomer fibers and between them, which also explains the higher anion conductivity of the mat electrospun at 20% RH_ES_.

## 4. Conclusions

In this study, anion-conducting fiber mats were prepared by electrospinning (ES) at different relative humidity (RH_ES_) values. The ionomer nanofibers’ morphology, anion conductivity, and water uptake (WU) were measured. Formation of branched thin fibers was observed in mats prepared at RH_ES_ 20% and 30%. The mechanism of branching as a function of RH_ES_ in ES of anion-conducting material was discussed. It was shown that ionomer fiber diameter distribution is significantly affected by the RH_ES_ up to RH = 40%. Above this value, the effect of RH_ES_ on the fiber diameter distribution was marginal. 

The results of WU and anion conductivity measurements showed that the ionomer mat electrospun under the lowest RH_ES_ (20%) gained the highest WU (24%) and TP conductivity (4.4 mS cm^−1^) at RH = 90%. These results are associated with the large fibers’ diameter and their high water capacity, which gives rise to higher anion conductivity. 

The preparation of anion-conducting nanofibers prepared by ES process can be attractive for numerous applications including AEMFCs. Therefore, understanding the interplay between process conditions such as relative humidity during electrospinning and the nanofibers’ structure, WU, as well as anion conductivity is important for the future design of these devices with tailored properties. This understanding is critical for effective water management within these future devices.
